# Neuroprotective Effects of Protocatechuic Aldehyde against Neurotoxin-Induced Cellular and Animal Models of Parkinson’s Disease 

**DOI:** 10.1371/journal.pone.0078220

**Published:** 2013-10-18

**Authors:** Xin Zhao, Shenyu Zhai, Ming-Sheng An, Yue-Hua Wang, Ying-Fan Yang, Hui-Qi Ge, Jin-Hao Liu, Xiao-Ping Pu

**Affiliations:** 1 National Key Research Laboratory of Natural and Biomimetic Drugs, Peking University, Beijing, P. R. China; 2 Department of Molecular and Cellular Pharmacology, School of Pharmaceutical Sciences, Peking University, Beijing, P. R. China; 3 Department of Neurobiology, Duke University Medical Center, Durham, North Carolina, United States of America; 4 Beijing Key Laboratory of Drug Target Identification and Drug Screening, Institute of Materia Medica, Chinese Academy of Medical Sciences & Peking Union Medical College, Beijing, P.R. China; Hertie Institute for Clinical Brain Research and German Center for Neurodegenerative Diseases, Germany

## Abstract

Protocatechuic aldehyde (PAL) has been reported to bind to DJ-1, a key protein involved in Parkinson’s disease (PD), and exerts potential neuroprotective effects via DJ-1 in SH-SY5Y cells. In this study, we investigated the neuroprotective pharmacological effects of PAL against neurotoxin-induced cell and animal models of PD. In cellular models of PD, PAL markedly increased cell viability rates, mitochondrial oxidation-reduction activity and mitochondrial membrane potential, and reduced intracellular ROS levels to prevent neurotoxicity in PC12 cells. In animal models of PD, PAL reduced the apomorphine injection, caused turning in 6-OHDA treated rats, and increased the motor coordination and stride decreases in MPTP treated mice. Meanwhile, in an MPTP mouse model, PAL prevented a decrease of the contents of dopamine (DA) and its metabolites in the striatum and TH-positive dopaminergic neuron loss in the substantia nigra (SN). In addition, PAL increased the protein expression of DJ-1 and reduced the level of α-synuclein in the SN of MPTP lesioned mice. PAL also increased the spine density in hippocampal CA1 neurons. The current study demonstrates that PAL can efficiently protect dopaminergic neurons against neurotoxin injury *in vitro* and *in vivo*, and that the potential mechanisms may be related to its effects in increasing DJ-1, decreasing α-synuclein and its growth-promoting effect on spine density.

## Introduction

Parkinson’s disease (PD) is one of the most common neurodegenerative diseases affecting the nervous system, and is frequently observed among the elderly population. The incidence of PD is increasing year by year as the population is ageing, and it is expected that the incidence will double within the next two decades [1,2]. Multiple mechanisms have been found that lead to PD progression, including genetic factors, environmental factors, aging, mitochondrial dysfunction, oxidative stress and so on. DJ-1 was identified as a causal gene for a familial form of early onset PD, and has been shown to play roles in transcriptional regulation, mitochondrial function and the anti-oxidative stress reaction [3-7]. α-Synuclein is a substantive component of Lewy bodies, one of the pathological hallmarks of PD [8]. It is implicated in oxidative injury and mitochondrial dysfunction, which ultimately induce neurodegeneration and cell death [9].

Protocatechuic aldehyde (PAL) is a compound that exists in several types of Chinese herbs, such as the roots of *Salvia miltiorrhiza*, a herb widely used in traditional Chinese medicine. At present, PAL has been found to possess several biological activities, although the mechanisms underlying these activities are still unclear. PAL is known to inhibit the migration and proliferation of vascular smooth muscle cells [10] and can also inhibit lipopolysaccharide-induced human umbilical vein endothelial cell apoptosis via regulation of caspase-3 intravascular thrombosis [11]. These findings suggest a potential therapeutic role for PAL in the treatment of atherosclerosis. Meanwhile, it has been reported that PAL can potentially confer anti-fibrosis effects through the inhibition of hepatic stellate cell (HSC) proliferation and levels of transforming growth factor-β1, connective tissue growth factor, type I collagen and type III collagen in tumor necrosis factor-α stimulated HSCs [12]. It has also been reported that PAL possesses other biological activities, including anti-sepsis [13], inhibition of hepatitis B virus replication [14], and anti-inflammatory and antioxidant activities [15]. 

In our previous study [16], we found that PAL had DJ-1-dependent anti-oxidative activity. Our group studied DJ-1-binding compounds and assessed their binding activity by quartz crystal microbalance (QCM), and identified PAL as one of the DJ-1-binding compounds. Subsequent *in vitro* studies showed that PAL could protect SH-SY5Y cells from oxidative stress–induced cell death in a DJ-1-dependent manner. We also discovered that PAL could prevent superfluous oxidation of Cys106, an essential amino acid for the function of DJ-1. These results demonstrated that PAL exerts potential neuroprotective effects via DJ-1 and could be a potential pharmaceutical reagent for PD.

The aim of the present study is to further investigate the neuroprotective effects of PAL using other PD cell models induced by H_2_O_2_ or 6-hydroxydopamine (6-OHDA) in PC12 cells, and PD rat or mice models respectively induced by 6-OHDA or 1-Methyl-4-phenyl-1,2,3,6-tetrahydropyridine (MPTP), in order to determine whether PAL could be a potential pharmaceutical reagent for PD. On this basis, in order to further elucidate its potential mechanisms, the effects of PAL treatment on DJ-1, α-synuclein protein expression and its growth-promoting property in hippocampal CA1 neurons were also studied.

## Materials and Methods

### 1: Ethics Statement

In the present study, all experiments were performed under the guidelines of the Experimental Laboratory Animal Committee of Peking University Health Science Center and were in strict accordance with the principles and guidelines of the National Institutes of Health Guide for the Care and Use of Laboratory Animals. The Animal Care and Use Committees of Peking University Health Science Center approved all animal care and experimental protocols. All surgery was performed under anesthesia, and all efforts were made to minimize animal suffering.

### 2: Materials

PAL was purchased from J&K Scientific Ltd. (Beijing, China), and HPLC analysis showed that its purity was greater than 98%. Edaravone Injection was the product of Simcere Pharmaceutical Group (China). Selegiline Hydrochloride was the product of Nanjing green leaves from Cisco Pharmaceutical Co., Ltd. Poly-L-lysine, trypsin, 3-(4,5-dimethylthiazol-2-yl)-2,5-diphenyltetrazolium bromide (MTT), MPTP and 6-OHDA were purchased from Sigma (St Louis, MO, U.S.A.). All other reagents were of analytical grade.

### 3: Cell culture and viability assays

PC12 cells were purchased from the Cell Resource Center, School of Basic Medicine, Peking Union Medical College, China. The cells were cultured in F12K with L-glutamine (292 mg/l) medium containing 5% fetal bovine serum, 15% equine serum, 100 U/ml penicillin, and 100 mg/ml streptomycin in a 5% CO_2_ humidified atmosphere at 37 °C.

PC12 cells were incubated in 96-well microplates (2×10^5^ cells / well in 100 µl) for 24 h. The cells were then pretreated with various concentrations of PAL (dissolved in phosphate buffered saline (PBS) for 6 h, and treated with 250 µM H_2_O_2_ for 1 h or 200 µM 6-OHDA for 24 h at 37 °C, respectively. The controls were exposed to the same solvent. Cell viability was measured using the MTT assay [17].

### 4: Determination of intracellular reactive oxygen species (ROS) levels

Intracellular ROS levels were measured using the 2, 7-dichlorofluorescein-dictate (H_2_DCFH-DA) staining method. After incubation with 6-OHDA, cells were loaded with 10 µM DCFHDA for 30 min at 37 °C in the dark. The fluorescence intensity of DCF was measured at an excitation wavelength of 495 nm and emission wavelength of 530 nm on a microplate reader.

### 5: Measurement of mitochondria oxidation-reduction (REDOX) activity by resazurin

After treatment, resazurin at a final concentration of 5 µM was added into the wells and the fluorescence intensity was examined at an excitation of 530 nm and an emission of 590 nm. The plate was incubated for another 60 min and then fluorescence was measured. The changing rate was represented as (F60 - F0) / F0*100%. F60, F0 refer to the fluorescence at 60 min and 0 min, respectively.

### 6: Measurement of mitochondrial membrane potential

The fluorescent probe JC-1 (5,5′,6,6′-tetrachloro-1,1′,3,3′-tetraethylbenzimidazolylcarbocyanine iodide) was used to estimate mitochondrial membrane potential (MMP). JC-1 is sensitive to MMP, and the changes in the ratio between aggregate (red) and monomer (green) fluorescence can provide information regarding the MMP. After treatment, the culture medium was removed and loaded with JC-1 for 15 min at 37 °C in the dark. After two more rinses with Hank’s solution, the fluorescence intensity of the red/green ratio was determined on a Spectramax M5 microplate reader (Molecular Devices, Sunnyvale, CA, USA) at an excitation of 490 nm and emission of 530 nm (green fluorescent monomers) and 590 nm (red fluorescent aggregates) respectively.

### 7: Animals and treatment

Adult male Sprague-Dawley rats weighing 250-300 g and C57BL/6 mice weighing 20–25 g were purchased from the Laboratory Animal Center of Peking University Health Science Center (Beijing, China). Purchased rats and mice met the approval of the local animal committee with confirmation number: SCXK(JING)2011-0012. Animals were housed under standard conditions (22±2 °C) with food and water available *ad libitum*. 

**Figure 1:**
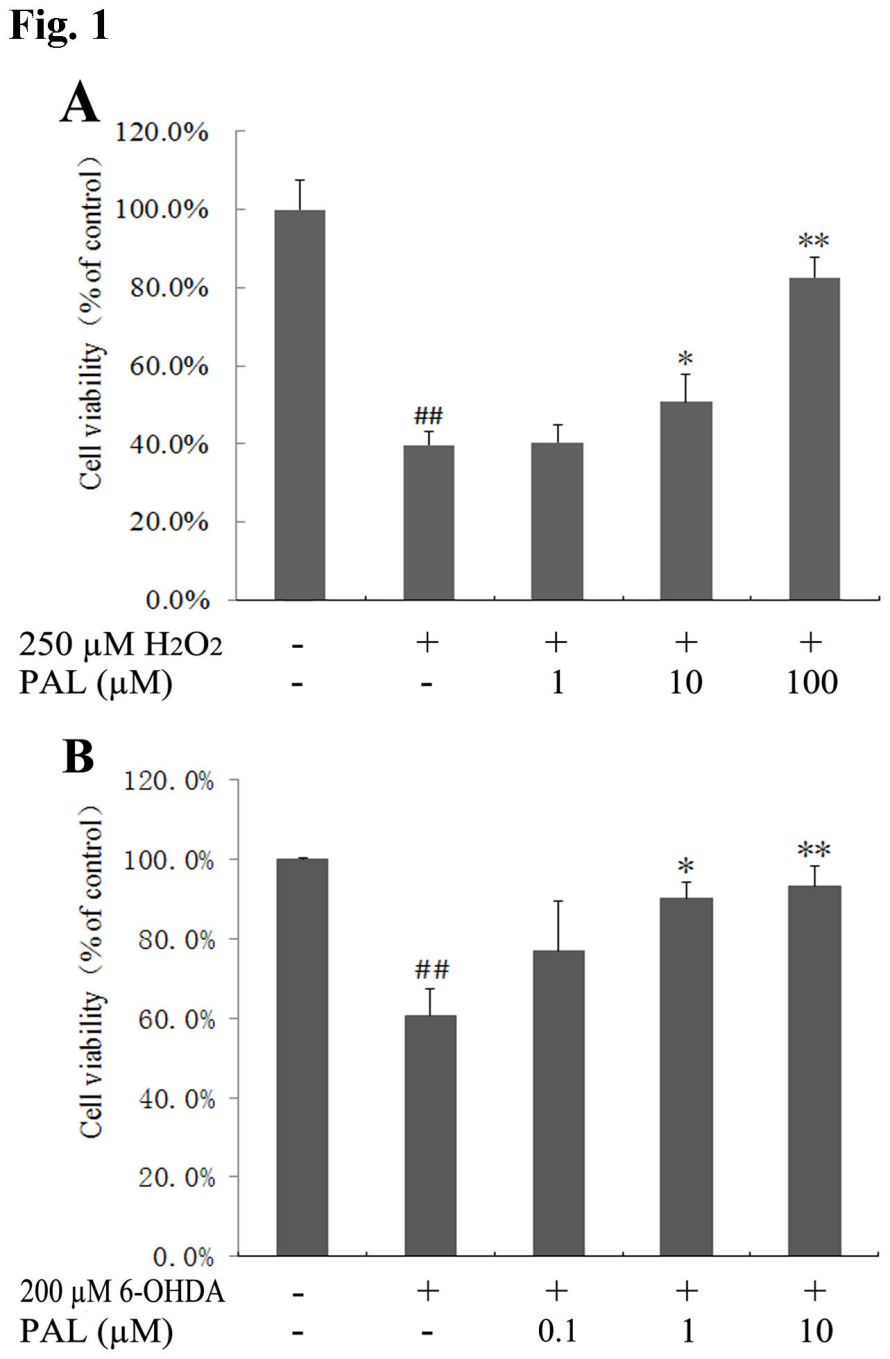
Pre-treatment with PAL improves cell viability after administration of H_2_O_2_ or 6-OHDA. (A) After 6 h PAL pre-treatment, cells were treated with 250 µM H_2_O_2_ for 1 h. (B) Cells were treated with 200 µM 6-OHDA for 24 h after pre-treatment with PAL for 6 h. Data represent the mean ± SEM (n = 3) from three independent experiments. Statistical analyses were performed using one-way ANOVA. ∗ *P* < 0.05, ** *P* < 0.01, compared with the H_2_O_2_ or 6-OHDA group. ## *P* < 0.01, compared with the control group.

#### 7.1: 6-OHDA-induced PD rat model

 Rats were randomly divided into the following 5 groups: sham control group, 6-OHDA group, low and high PAL dose groups (treated with PAL at doses of 20 and 40 mg/kg, respectively), and the selegiline group (positive-control, treated with selegiline at dose of 7.5 mg/kg). The stereotaxic microinjection of 6-OHDA was carried out in accordance with a previously published method [18]. Rats were anesthetized (sodium pentobarbital, 50 mg/kg, i.p.) and immobilized in a stereotaxic frame. All rats, with the exception of those in the sham control group, were injected with 6-OHDA (6.0 μg, final concentration 6 mM) to a final volume of 4 μl of PBS, while rats in the sham control group were injected with same volume of PBS. Stereotaxic coordinates, relative to bregma, were: AP = −4.8 mm, ML = −1.8 mm, DV = −7.8 mm, and the toothbar was set at −2.4 mm. After 28 days, rats in the low and high PAL dose groups were administered with PAL (20 mg/kg or 40 mg/kg) orally (*per os* (*p.o.*)) every 24 h for 28 consecutive days. Rats in the sham control and 6-OHDA groups were administered with same volume of normal saline.

**Figure 2:**
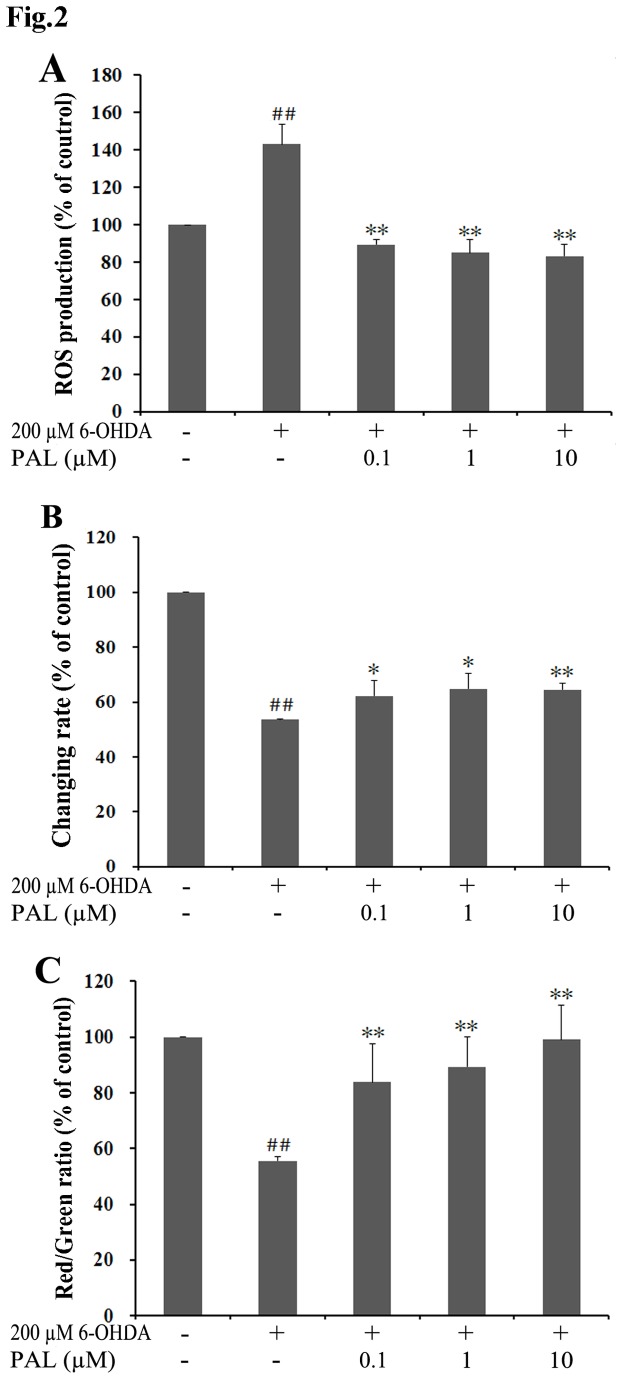
Pre-treatment with PAL preventes 6-OHDA-induced ROS production and mitochondrial damage in PC12 cells. (A) Effect of PAL on generation of ROS measured by incubation with H2DCF-DA. (B) Effect of PAL on REDOX activity detected by resazurin. (C) Effect of PAL on mitochondria membrane potential assayed by JC-1. Cells were treated with 200 µM 6-OHDA for 24 h after pre-treating with PAL for 6 h. Data represent the mean ± SEM (n = 3) from three independent experiments. Statistical analyses were performed using one-way ANOVA. ∗ *P* < 0.05, ** *P* < 0.01, compared with the 6-OHDA group. ## *P* < 0.01, compared with the control group.

#### 7.2: MPTP induced PD mice model

In behavioral and Western blotting experiments, after being allowed to acclimatize in our facilities for 7 days, mice were randomly divided into the following 6 groups, containing 12 mice per group: vehicle control group (an equal volume of normal saline); MPTP model group (pre-treatment with an equal volume of saline); low, moderate and high PAL dose groups (pre-treatment with PAL at doses of 7.5, 15 and 30 mg/kg, respectively); selegiline group (positive-control, pre-treatment with selegiline at dose of 15 mg/kg). In HPLC and immunohistochemistry experiments, mice were randomly divided into the following 4 groups, containing 9 mice per group: vehicle control group; MPTP model group; PAL group (pre-treatment with PAL at doses of 5 mg/kg); selegiline group (positive-control, pre-treatment with selegiline at dose of 15 mg/kg). All groups were orally administered with the respective pre-treatment compounds (*per os* (*p.o.*)) every 12 h for 14 consecutive days. With the exception of the vehicle control group, mice were treated with a conventional MPTP paradigm: MPTP (30 mg/kg, dissolved in saline) was intraperitoneally injected (*i.p.*) once a day for 5 consecutive days from day 10 to day 14. For all groups, MPTP was injected intraperitoneally 1 h following administration of the pre-treatment of PAL or selegiline. Control animals were injected with saline under the same regimen [19,20]. 

### 8: Behavior test

#### 8.1: Apomorphine-induced rotational behavior of the rats

One day after the final PAL administration, rats belonging to each group received a single intraperitoneal injection of apomorphine (1 mg/kg in normal saline). Rotational asymmetry was scored for 30 min continuously and complete contralateral rotations were scored [18].

#### 8.2: The behavior test of mice

Behavioral experiments, including rotarod performance tests and gait tests, were conducted 1 d after the final MPTP injection [21].

 The rotarod performance test was used to assess sensorimotor coordination of the mice [19]. The time for which each mouse remained on the rotating bar was recorded over 3 tests for each mouse at 3-min intervals. Data are presented as the mean time on the rotating bar over the 3 test trials.

The gait test was performed in a vertical grid apparatus as described in the literature [22]. Before the formal experiment, the mice were acclimatized to the vertical grid 3 times a day over two successive days. The trials were then made and videotaped after MPTP administration. The videos were replayed for analysis of the total number of hind limb steps. Data are presented as the mean number of steps over the 3 trials. 

### 9: Dopamine (DA) and its metabolites measurements by HPLC

Three days after the final MPTP treatment, 6 mice from each group were sacrificed and their brains were quickly removed and placed on ice. Their striatums were dissected out and weighed. The contents of DA and its metabolites 3,4-dihydroxyphenylacetic acid (DOPAC) and homovanillic acid (HVA) were assayed by HPLC with electrochemical detection (ECD). HPLC analysis was performed based on previously described methods [18].

### 10: Tyrosine hydroxylase (TH) immunohistochemistry

Three days after the final MPTP treatment, 3 mice from each group were subjected to perfusion of PBS through the aorta followed by cold 4% paraformaldehyde, under deep anesthesia. TH-immunohistochemistry on SN sections, including microphotograph capture and data analysis, were performed in full accordance with the previously described protocols [18]. 

### 11: Western blotting

The substantia nigra (SN) of mice were dissected 3 d after the final MPTP treatment. Protein in the SN was obtained and assayed based on previously described methods [19]. Antibodies directed against DJ-1 [kindly provided by Prof. Hiroyoshi Ariga (Hokkaido University, Japan), 1:6000] or α-synuclein (Santa Cruz, 1:600) were used as the primary antibody. Horseradish peroxidase-conjugated IgG (anti-mouse or rabbit, Santa Cruz) was used as a secondary antibody. To ensure equal protein loading, membranes were stripped and immunostained for actin using an anti-β-actin antibody (1:1000, Santa Cruz) as the primary antibody. The band intensities were quantified using Quantity One software (Bio-Rad, USA). Western blotting was repeated 3 times for each sample, with duplicate measurements for each blot.

### 12: Growth-promoting effect on spine density

#### 12.1: Hippocampal slice culture and treatments

Organotypic hippocampal slice cultures were prepared from P6-7 rats, as described previously [23]. Rats were decapitated after anesthesia and their brains removed. Hippocampi were dissected out and coronal slices of 350 μm thickness were cut by a McIlwain tissue chopper (Campden). After 11-13 days *in vitro*, CA1 pyramidal neurons were transfected with mEGFP using a gene-gun (Bio-Rad) [24]. One day after transfection, slices were incubated with PAL (20 μM) or vehicle (0.1% dH_2_O) for 48 h before imaging.

#### 12.2: Image acquisition and analysis

CA1 pyramidal neurons expressing mEGFP were imaged at room temperature using a custom-built two-photon microscope with a Ti:sapphire pulsed laser tuned to 920 nm (Spectra-Physics). Images were taken at high magnification (60X water-immersion objective) and magnified further using a 16X zoom. Z-stacks were acquired at 0.67-μm intervals. Apical secondary and tertiary dendrites 50-200 μm away from soma were randomly chosen for imaging (Image analysis was performed with the investigator blinded to the treatments used). Spine density was quantified manually using ImageJ (NIH) with the Cell Counter plug-in. Spine counting did not include protrusions in the Z-axis that could not be identified unambiguously on z-stack projection images. The total number of spines was divided by the total length of dendritic segments on which the counted spines reside.

### 13: Statistical analysis of the data

The data are expressed as means ± SEM. Statistical comparisons were performed using one-way ANOVA. A *P*-value less than 0.05 was considered to be a significant difference.

## Results

### 1: Effects of PAL on cell viability in PC12 cells exposed to H_2_O_2_ or 6-OHDA

Two cellular models were employed to verify the results of our previous study [16]. The protective effects of PAL were evaluated on MTT reduction in cultured PC12 cells. Under both treatments (1 h of H_2_O_2_ or 24 h of 6-OHDA), the number of viable cells was significantly decreased. However, when samples were pretreated with various concentrations of PAL for 6 h, the cell viability rates markedly increased under both conditions (Figure 1A and 1B), especially in the 6-OHDA group, where the increase was in a concentration-dependent manner (Figure 1B). 

**Figure 3:**
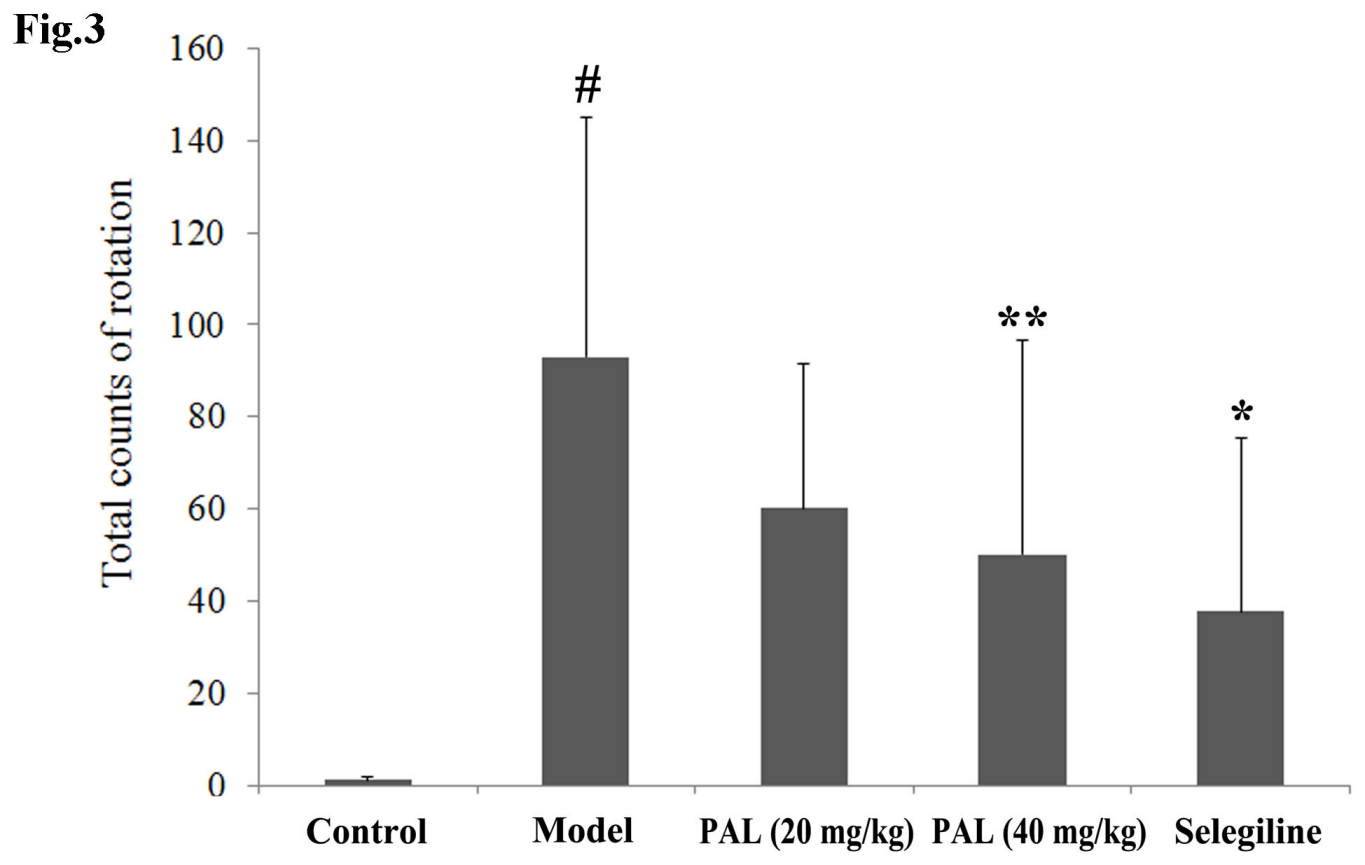
Effects of PAL on apomorphine-induced rotations in the 6-OHDA-induced rats model of PD. Data are expressed as means ± SEM (n = 5). Statistical analyses were performed using one-way ANOVA. *P < 0.05, **P < 0.01 compared with the model (6-OHDA) group; #*P* < 0.05 compared with the sham control group.

### 2: Effects of PAL on intracellular ROS levels REDOX activity and MMP in PC12 cells exposed to 6-OHDA

The effects of PAL on intracellular ROS levels were detected by DCF fluorescence (Figure 2A). REDOX activity was detected by resazurin (Figure 2B). Changes in MMP were measured by determining the red/green fluorescence ratio of JC-1 (Figure 2C). As shown in Figure 2, treatment of cultures with 6-OHDA for 24 h resulted in a significant increase of intracellular ROS levels compared with the control group (*P* < 0.01). Meanwhile, treatment with 6-OHDA also led to an obvious decrease of REDOX activity and MMP (*P* < 0.01, vs control group). However, the changes induced by 6-OHDA were attenuated by treatment with 0.1, 1 and 10 μM PAL (*P* < 0.05 or *P* < 0.01, vs 6-OHDA group, respectively). These results indicate that the co-incubation of PC12 cells with PAL effectively prevents the 6-OHDA-induced production of ROS and mitochondrial damage.

**Figure 4:**
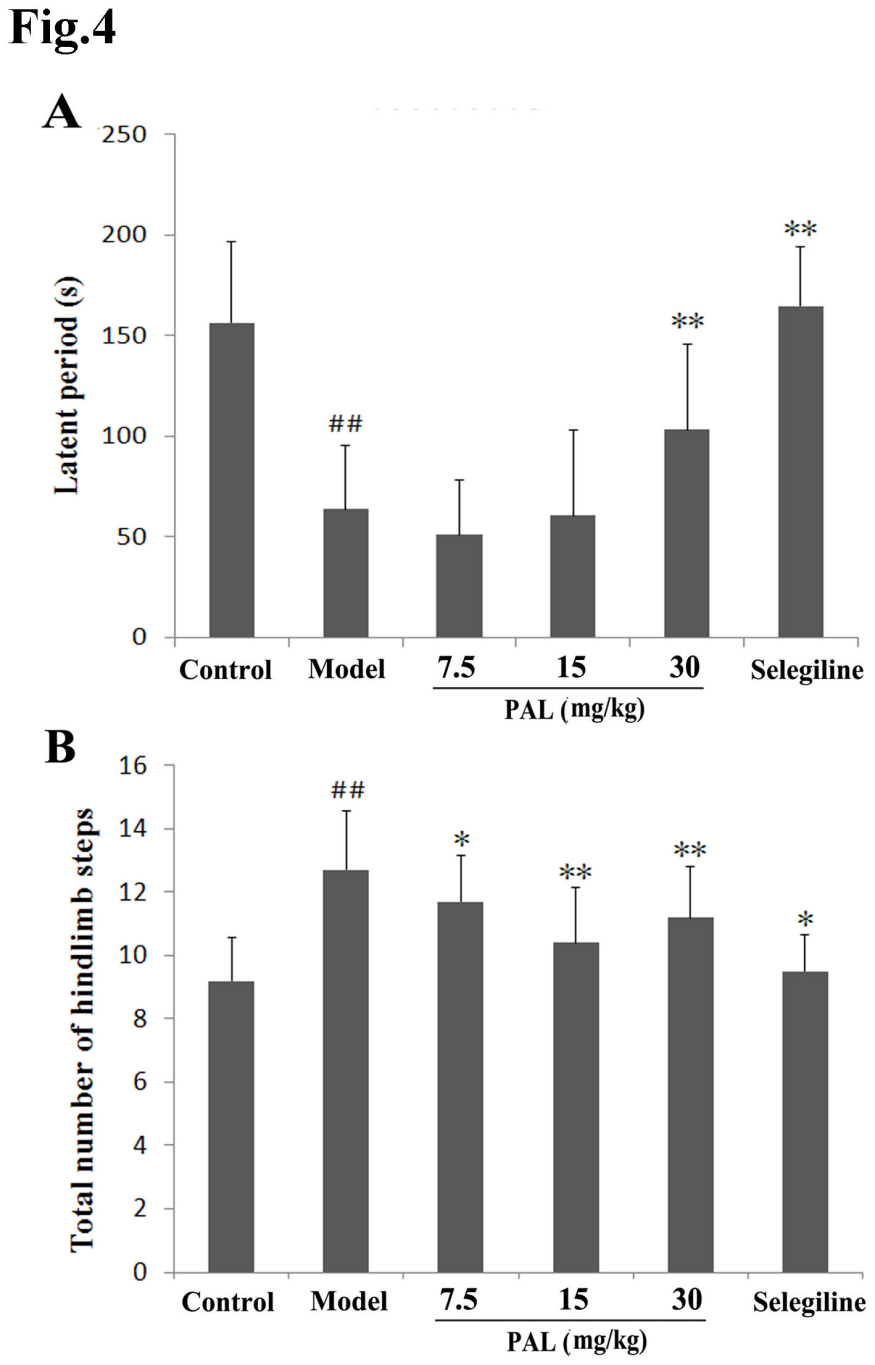
Effects of PAL on motor behavioral in the MPTP mice model of PD. (A) The time period on the rotarod. (B) Total number of hind limb steps. Data are expressed as means ± SEM (n = 12). Statistical analyses were performed using one-way ANOVA. *P < 0.05, **P < 0.01 compared with the model (MPTP) group; ##*P* < 0.01 compared with the control group.

### 3: Apomorphine-induced rotational behavior of the rat model

The effects of PAL were first detected in the 6-OHDA-induced rat model, one of the classic PD animal models. At 28 days of PAL treatment, rats in each group were assessed for rotational behavior (Figure 3). Similar to our previous reports [18], an apomorphine injection caused marked turning in 6-OHDA group rats (*P* < 0.05 vs. sham control group). Rotations in the high dose PAL group (40 mg/kg) and positive control group (selegiline) were seen to significantly decrease (*P* < 0.01 or *P* < 0.05 vs. 6-OHDA group, respectively).

**Figure 5:**
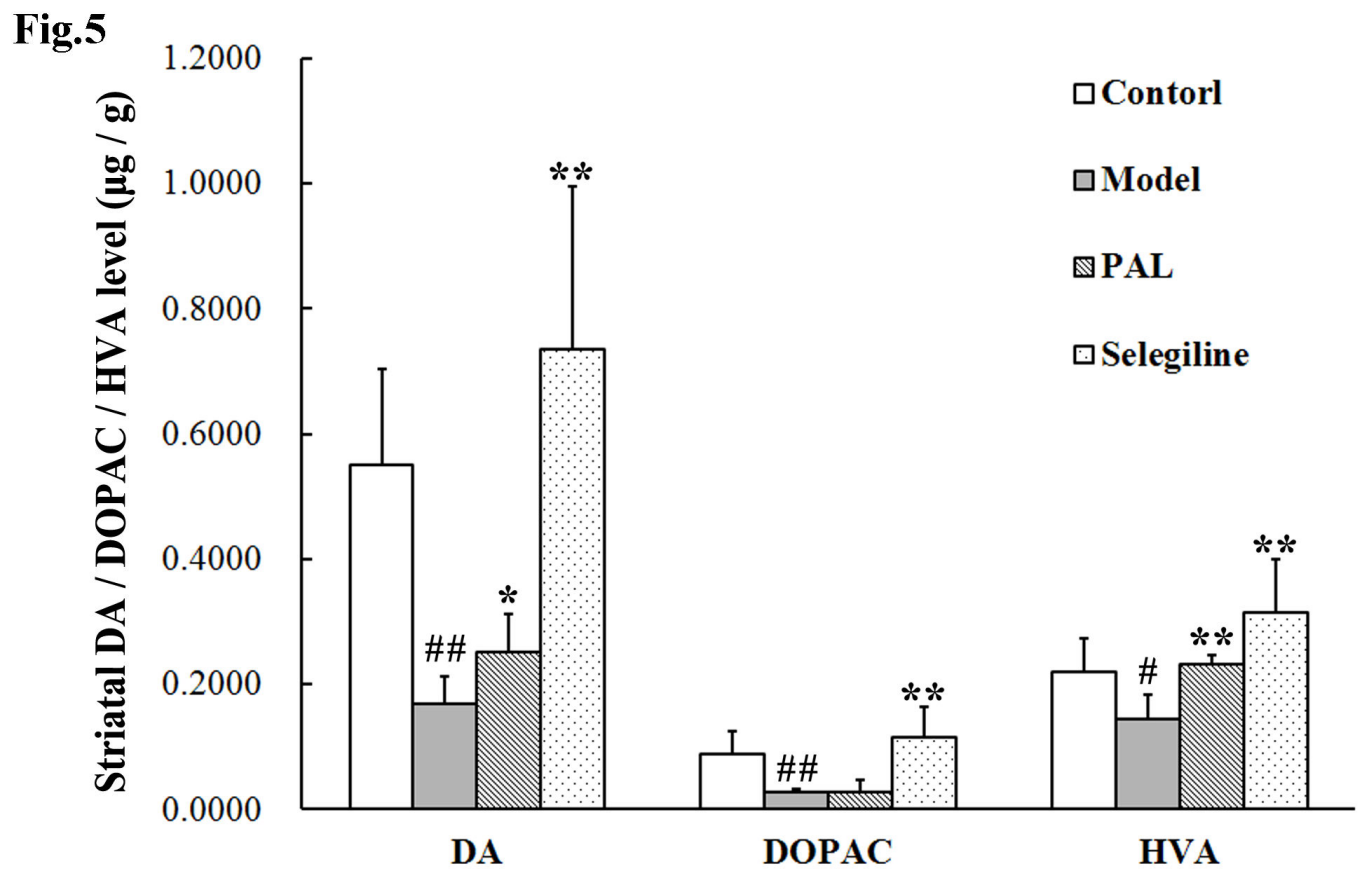
Effects of PAL on DA and its metabolites in the striatum of MPTP mice model of PD. Data are expressed as means ± SEM (n = 6). Statistical analyses were performed using one-way ANOVA. *P < 0.05, **P < 0.01 compared with the model (MPTP) group; #*P* < 0.05, ##*P* < 0.01 compared with the control group.

### 4: Motor behavioral test of MPTP induced PD mice model

Figure 4A and B show results for the rotarod and gait tests, respectively. In the rotarod test, the latent period representing the time for which the mice remained on the bar was significantly reduced in the MPTP model group relative to the control group (*P* < 0.01). In the groups pretreated with high doses of PAL, the latent period was increased relative to the MPTP model group (*P* < 0.01). In the gait test, MPTP administration resulted in a significant increase in the total number of hind limb steps relative to the untreated mice (control group). All three PAL doses significantly prevented this increase (*P* < 0.05 or *P* < 0.01). 

**Figure 6:**
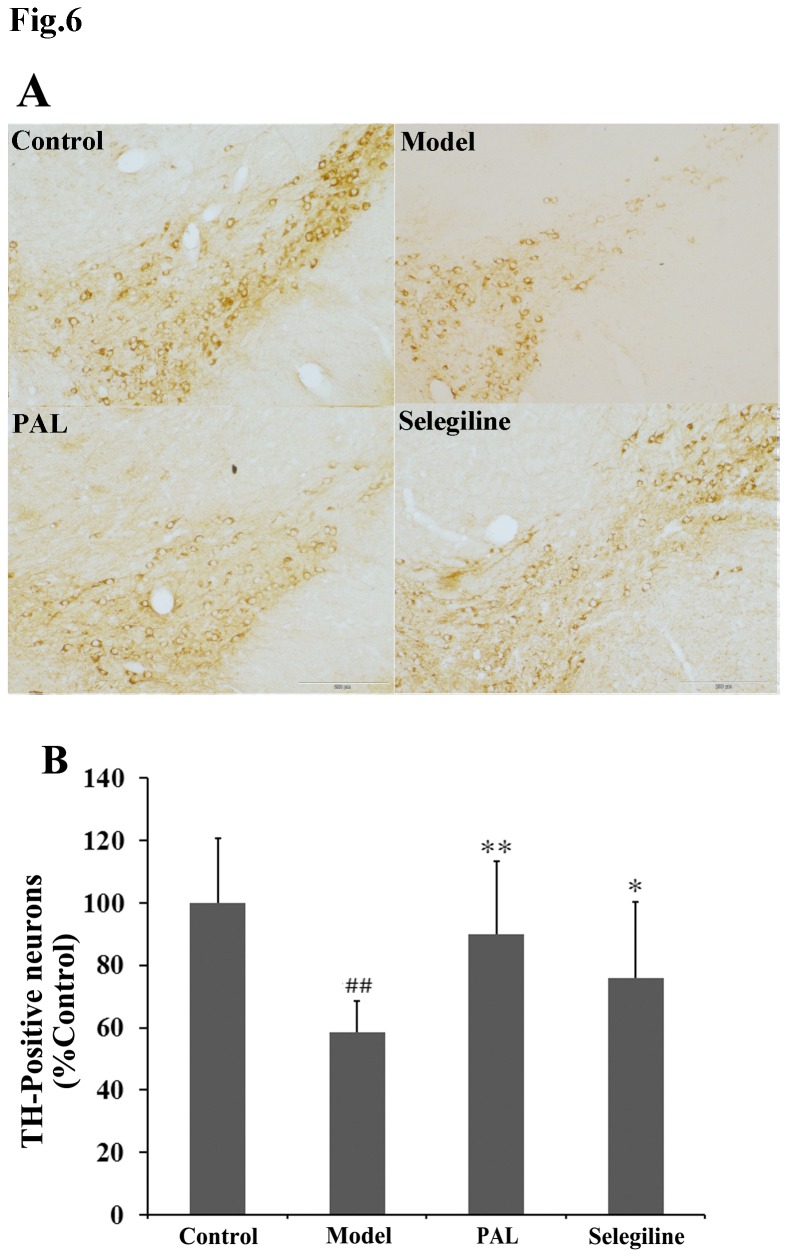
Effects of PAL on dopaminergic neuronal death in the SN. (A) TH immunohistochemistry of the right SN. (B) TH-positiveneurons count. Values are presented as means (% of control) ± SEM of 3 mice per group and 4 sections per mouse. Statistical analyses were performed using one-way ANOVA. *P < 0.05, **P < 0.01 compared with the model (MPTP) group; ##*P* < 0.01 compared with the control group.

### 5: Striatal DA and its metabolites contents

The effects of PAL on the levels of DA and its metabolites in the striatum of MPTP-induced PD model mice are shown in Figure 5. DA, DOPAC and HVA contents in the striatum of MPTP-lesioned mice were significantly lower than those in the control group (*P* < 0.01, *P* < 0.01, *P* < 0.05 vs. control group, respectively). After administration of PAL, the striatal DA and HVA content of the PAL group was higher than in the model group (*P* < 0.05 or *P* < 0.01 vs. model group, respectively), and the striatal DOPAC content exhibited no statistically significant increase. 

### 6: TH immunohistochemistry

TH staining was performed to evaluate the survival of dopaminergic neurons. Morphological observations are shown in Figure 6A. In control mice, the cytoplasm and fibers of dopaminergic neurons were intensely stained and the cellular processes were evident. In contrast, mice in the MPTP model group showed a marked loss of DA-containing SN neurons, as few TH-positive cells were observed and the cellular processes were absent for most cells. PAL or selegiline administration resulted in an increase in TH-positive cells with similar cell morphology to the control group, suggesting PAL could protect dopaminergic neurons from MPTP neurotoxicity in mice. 

Consistent with the above cellular morphological observations, the average cell count in the MPTP model group was significantly reduced (*P* < 0.01 vs. control group). Loss of dopaminergic neurons was inhibited by pre-treatment of PAL (*P* < 0.01 vs. MPTP model group) (Figure 6B).

### 7: The expression of DJ-1 and α-synuclein in the SN of PD mice model

**Figure 7:**
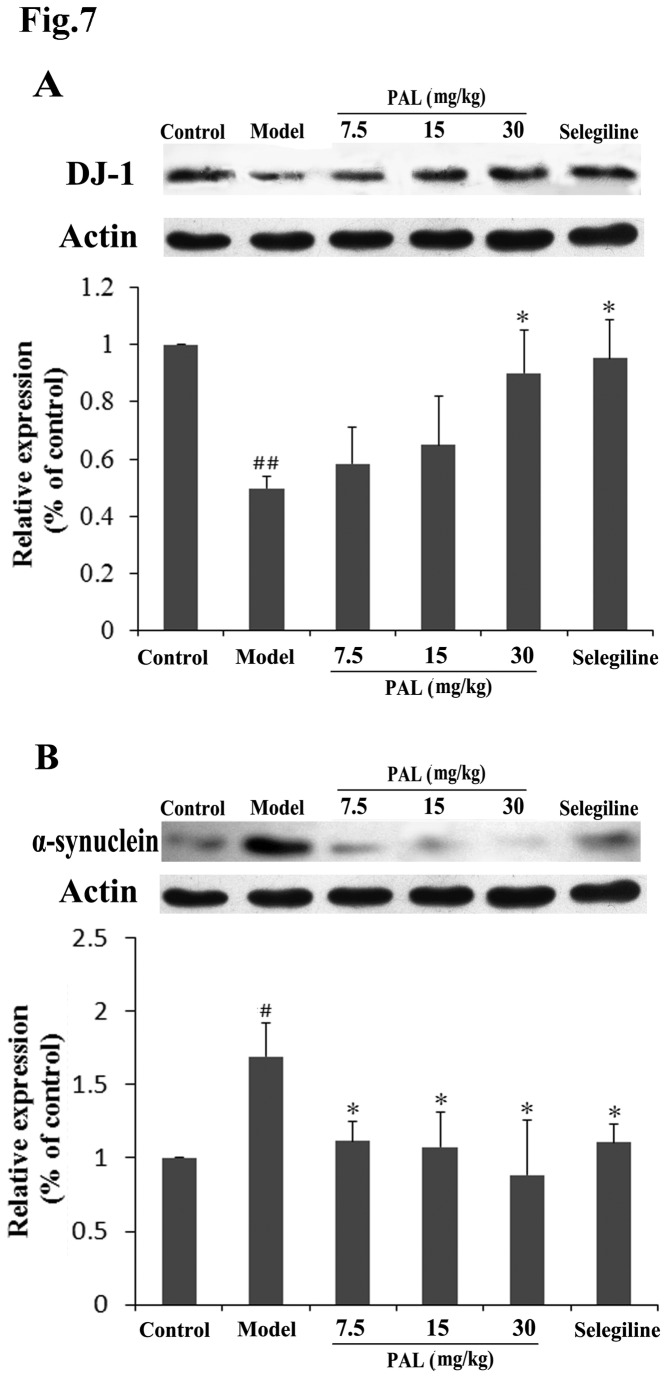
Effects of PAL on expression levels of DJ-1 and α-synuclein in the SN of mice model of PD. (A) PAL increases DJ-1 expression in the SN of PD mice model; (B) PAL reduces α-synuclein expression in the SN of PD mice model. The expression of DJ-1 and α-synuclein were measured by Western blotting. Data are expressed as means (% of control) ± SEM (n = 3). Statistical analyses were performed using one-way ANOVA. *P < 0.05, compared with the model (MPTP) group; #*P* < 0.05, ##*P* < 0.01, compared with the control group.

Both DJ-1 and α-synuclein play important roles in PD and have altered expression levels in MPTP-lesioned animals [25,26]. Furthermore, there is evidence that DJ-1 and α-synuclein are closely related [18,27,28]. Our previous study, however, did not examine the effects of PAL on these two proteins. Therefore, we selected these two proteins in order to conduct a preliminary study. The expression levels of DJ-1 and α-synuclein in the SN of the MPTP-lesioned mice were measured using Western blot analysis (Figure 7A and B). The level of DJ-1 in MPTP-lesioned mice was significantly reduced (*P* < 0.01, compared with control group), leading to an upregulation of α-synuclein (*P* < 0.05, compared with control group). The administration of PAL or selegiline was able to prevent these changes. After administration of a high dose of PAL (30 mg/kg) or selegiline, the levels of DJ-1 in the SN were significantly higher than in the MPTP model group (*P* < 0.05, compared with MPTP model group) (Figure 7A). Consistently, in the SN of the mice pretreated with various concentrations of PAL or selegiline, the levels of α-synuclein were lower than in the MPTP model group (all *P* < 0.05, compared with MPTP model group) (Figure 7B). 

### 8: Growth-promoting effects on spine density of PAL

**Figure 8:**
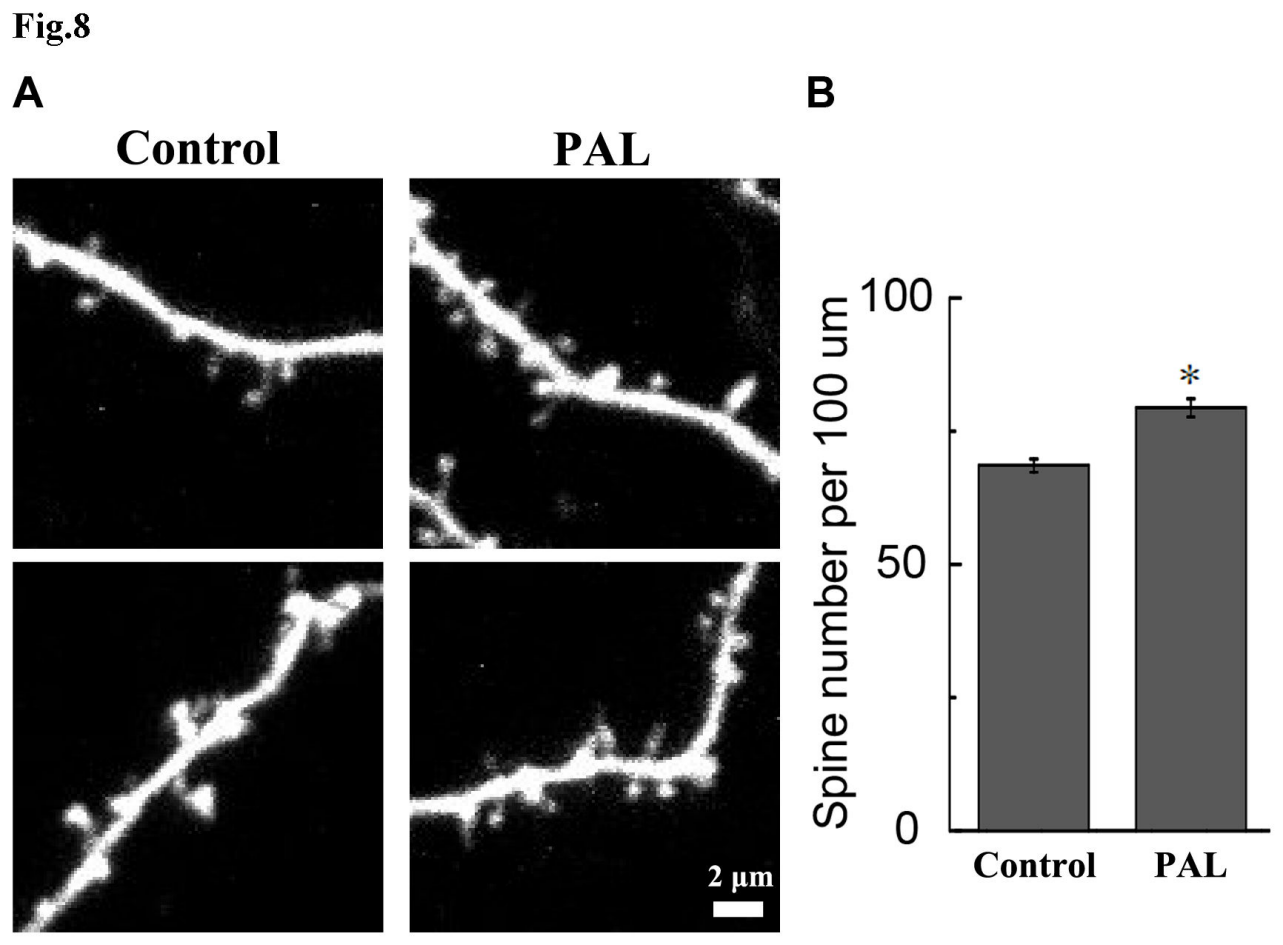
Effects of PAL on dendritic spine density in hippocampal CA1 neurons. (A) Images of secondary/tertiary apical dendrites of mEGFP-expressing t hippocampal CA1 neurons from vehicle or PAL treated slices. Scale bar, 2 µm. (B) PAL treatment resulted in an increase in spine density compared with control. The total number of spines was divided by the total length of dendritic segments on which counted spines resided. Data are represented as means ± SEM (n = 3). Statistical analyses were performed using one-way ANOVA. *P < 0.05, compared with the control.

To study the growth-promoting properties of PAL, we assessed its effects on spine density in apical secondary and tertiary dendrites of mEGFP-expressing CA1 neurons. Gene-gun transfection yielded sparse fluorescent labeling of CA1 neurons that allowed for imaging of the fine structure of dendrites against a dark background (Figure 8A). Application of PAL (20 μM) in the culture medium for 48 h resulted in a 14% increase in spine density compared with control (*P* < 0.05, Figure 8B). Specifically, the spine density in PAL-treated neurons was 79.38 ± 1.72 spines per 100 µm of dendritic length (6 cells from 3 batches of cultures), whereas the density in the control was 68.53 ± 1.25 spines per 100 µm of dendritic length (5 cells from the same batches of cultures).

## Discussion

 At present, PD drug treatment strategies are still not satisfactory. The main treatments for PD are symptomatic therapies, and L-dopa remains the most effective drug for treating symptoms of PD. However, some studies have shown that L-dopa is neurotoxic and that chronic L-dopa usage could induce motor and mental complications in the majority of patients [29,30]. Therefore, there is still an urgent need for new drugs for PD therapy, and particularly for drugs with neuroprotective effects. 

PAL is a traditional Chinese medicine compound and has been found to have several biological activities, including anti-atherosclerosis [10,11], anti-fibrosis [12], anti-sepsis [13], anti-inflammatory and antioxidant [15] activities. PAL is also an important compound in pharmaceutical preparations containing the roots of *Salvia miltiorrhiza*. The PAL content has been used as an active ingredient quality standard for a number of pharmaceutical preparations, including Sanbao capsules (Pharmacopoeia of The People’s Republic of China) and Danshen injections (Drug Standards of Ministry of Health). 

Our previous studies demonstrated that PAL could enter cells through the cell membrane (unpublished result) and has potential neuroprotective effects against H_2_O_2_-induced cell death in SH-SY5Y cells in a DJ-1-dependent manner [16]. We therefore carried out comprehensive studies *in vitro* and *in vivo* in order to determine whether PAL could be a potential pharmaceutical reagent or a candidate for PD therapy.

In our study, the protective effects of PAL were first verified *in vitro* using two other PD cellular models induced by H_2_O_2_ or 6-OHDA in PC12 cells. H_2_O_2_ and 6-OHDA are neurotoxins commonly used in PD cellular models [31,32]. PC12, the rat pheochromocytoma cell line, has been utilized as a neuron-like cell suitable as a dopaminergic nerve cell model for PD research [33]. Our data show that pre-treatment of PC12 cells with PAL can markedly increase the cell viability rates in H_2_O_2_ or 6-OHDA-induced neuronal damage. Moreover, 6-OHDA generates ROS, inhibits complexes I and IV of the mitochondrial respiratory chain and induces apoptosis in PC12 cells [34]. Pre-treatment with PAL could increase REDOX activity, MMP and reduce intracellular ROS levels to prevent 6-OHDA-induced mitochondrial damage and apoptosis in PC12 cells. These results are consistent with our previous studies and verify the neuroprotective effects of PAL in SH-SY5Y cells. 

On the basis of our results from *in vitro* experiments, *in vivo* studies were then performed. Neurotoxin-based models produced by 6-OHDA and MPTP administration are the most widely used toxic models. These two neurotoxins can be selectively accumulated in SN dopaminergic neurons, causing cellular dysfunction and death [35,36]. We chose the 6-OHDA rat model and MPTP mouse model to evaluate the neuroprotective pharmacological effects of PAL *in vivo*. In the present study, the results showed that 6-OHDA and MPTP could lead to specific behavioral defects. For instance, an apomorphine injection caused turning in 6-OHDA treated rats and reduced motor coordination and stride decreases in MPTP treated mice, while PAL pre-treatment was found to improve the behavioral performances. However, the seemingly large error bars obtained in these experiments may be due to individual differences between the animals in the ethological test, and other various factors that may influence the data. On the other hand, differences did exist in the effects of PAL on various behavioral indexes. We, therefore, added measurement of DA and its metabolites (Figure 5) and TH immunohistochemistry (Figure 6) in the MPTP model to further confirm the anti-PD effect of PAL in MPTP-induced mouse model. The results from HPLC showed that PAL inhibited the reduction of DA and its metabolite HVA contents in the striatum induced by MPTP. TH is the rate-limiting enzyme in dopamine biosynthesis and the marker for dopaminergic neurons. TH immunohistochemistry is often used to observe the survival of dopaminergic neurons. Our data showed that MPTP induced TH-positive dopaminergic neuron loss, and PAL pre-treatment could prevent this loss. In summary, these results suggest that PAL can protect dopaminergic neurons from 6-OHDA or MPTP damage *in vivo*, further demonstrating its potential as an anti-PD candidate or drug.

To further investigate the mechanisms underlying the protective actions of PAL, we studied the effects of PAL on expression of DJ-1 and α-synuclein in the SN of a PD mice model by Western blot analysis. In our previous study, we determined the DJ-1- binding property of PAL using QCM, and demonstrated that PAL protected neurons from oxidative damage in a DJ-1-dependent manner [16]. MPTP treatment decreased the expression level of DJ-1 in the striatum of the mice. Overexpression of DJ-1 renders dopaminergic neurons resistant to neurotoxic insults. Drugs that enhance DJ-1 gene expression are believed to neuroprotective for PD [25,37]. In the present study, similar changes were observed in the SN in the MPTP mice model. Our results showed that the expression of DJ-1 in the SN was decreased after MPTP treatment, but pre-treatment with PAL increased the level of DJ-1. Therefore, these results again suggest that DJ-1 plays a key role in the neuroprotective effect of PAL. We have previously demonstrated that PAL binds to DJ-1 and prevents its superfluous oxidation at Cys-106 [16]. It is very likely that PAL increases DJ-1 levels in the MPTP mice model through direct interaction and stabilization of DJ-1. However, other mechanisms, such as transcriptional upregulation, are also possible. α-Synuclein is the main structural component of Lewy bodies that is characteristic of both sporadic and familial PD [38]. It has been reported that α-synuclein in SN dopaminergic neurons is up-regulated following administration of MPTP [26,39]. Consistent with previous studies, we found that the expression of α-synuclein in the SN was increased in MPTP-lesioned mice, but pre-treatment with PAL markedly decreased the α-synuclein levels. These results indicate that α-synuclein might also be involved in the neuroprotective effects of PAL. In addition, it has been reported that overexpression of DJ-1 could inhibit mutant human α-synuclein protein aggregation [27,40,41], and DJ-1 inactivation may promote α-synuclein aggregation and the related toxicity [28]. Whether the reduction in α-synuclein induced by PAL is mediated by increased DJ-1 still needs to be explored further. In this paper, we focused on the anti-PD pharmacological effect of PAL, thus we only observed its influence on DJ-1 and α-synuclein expression at a macroscopic level. Although the mechanistic study of PAL is still preliminary and further investigations are required, this manuscript provides prospects for its future development.

With the exception of mechanisms related to anti-neurotoxin injury, we have demonstrated that exposure to PAL increases spine density in hippocampal CA1 neurons, suggesting a growth-promoting effect of the compound. Loss of dendritic spines and synaptic connections is strongly correlated with functional deficits in neurodegenerative diseases such as PD [42-44]. Restoration of spine density by pharmacological approaches has been shown to improve the behavioral efficacy of dopamine grafts in parkinsonian rats [45]. As a result, spine density restoration has been proposed as a potential therapeutic approach for treating neurodegenerative diseases [46]. Its benefits have been shown in animal models of PD [45]. The significance of the spine growth-promoting effect of PAL observed in the hippocampal neurons is three-fold. First, dementia, a frequent and debilitating symptom in advanced PD patients, has been strongly associated with several features of hippocampal degeneration, including hippocampus atrophy, axonal atrophy, increased Lewy body density, and increased Lewy neuritis [47-52]. Given the importance of the hippocampus in cognitive functions, neuronal degeneration and resultant impairment in the unidirectional flow of information in the hippocampus may underlie the dementia in PD patients. By strengthening the synaptic transmission in the hippocampus, PAL may provide a promising therapeutic approach for treating cognitive impairments associated with PD. Second, the density of spines is reduced on spiny neurons in basal ganglia both in neurotoxin-lesioned animal models of PD and in PD patients [42,53]. Furthermore, a multi-photon imaging study has revealed that DA depletion, through disinhibiting Cav1.3 channels, leads to spine loss in the striatopallidal medium spiny neurons (MSNs), thereby disconnecting these neurons from motor command brain regions [54]. Therefore, spine loss in MSNs may be a key step in the pathological progression of PD. Indeed, restoration of spine density has been proposed as a novel therapeutic approach for treating neurodegenerative diseases such as PD [46,55,56]. Regardless, the increase in spine density by PAL treatment suggests that PAL might be used to prevent or reverse spine loss in the MSNs and to alleviate motor symptoms in PD patients. Finally, it is known that the dendritic content and activity of mitochondria positively regulate spine density: enhanced mitochondrial activity leads to increased spine density whereas depletion of dendritic mitochondria leads to spine loss [57]. As suggested by our measurements of ROS generation and MMP (Figure 2), PAL protects or even improves mitochondrial function. Therefore, it is reasonable to ask if PAL treatment could lead to increased spine density. This seems to be the case: PAL treatment increased the dendritic spine density in CA1 pyramidal neurons by 14% (Figure 8). Although other possible mechanisms are yet to be ruled out, our results strongly suggest a model in which PAL increases spine density through strengthening mitochondrial function.

Due to the compound’s growth-promoting effects on spine density, we envision PAL as a potential treatment or candidate for PD or other neurodegenerative diseases. Notably, the effects of PAL on spine density were studied in the presence of growth factors present in the horse serum in the culture medium. Therefore, PAL could work independently of the neurotrophic factor therapy to treat neurodegenerative diseases. In the future, the effects of PAL on spine density should be further evaluated in PD animal models (since spines extending in the Z-axis of the image were excluded from analysis to minimize ambiguity, the spine density quantified in this study is an underestimation of the actual spine density. Therefore, the spine density of control we report here is lower than that reported in previous studies [58,59]).

**Figure 9:**
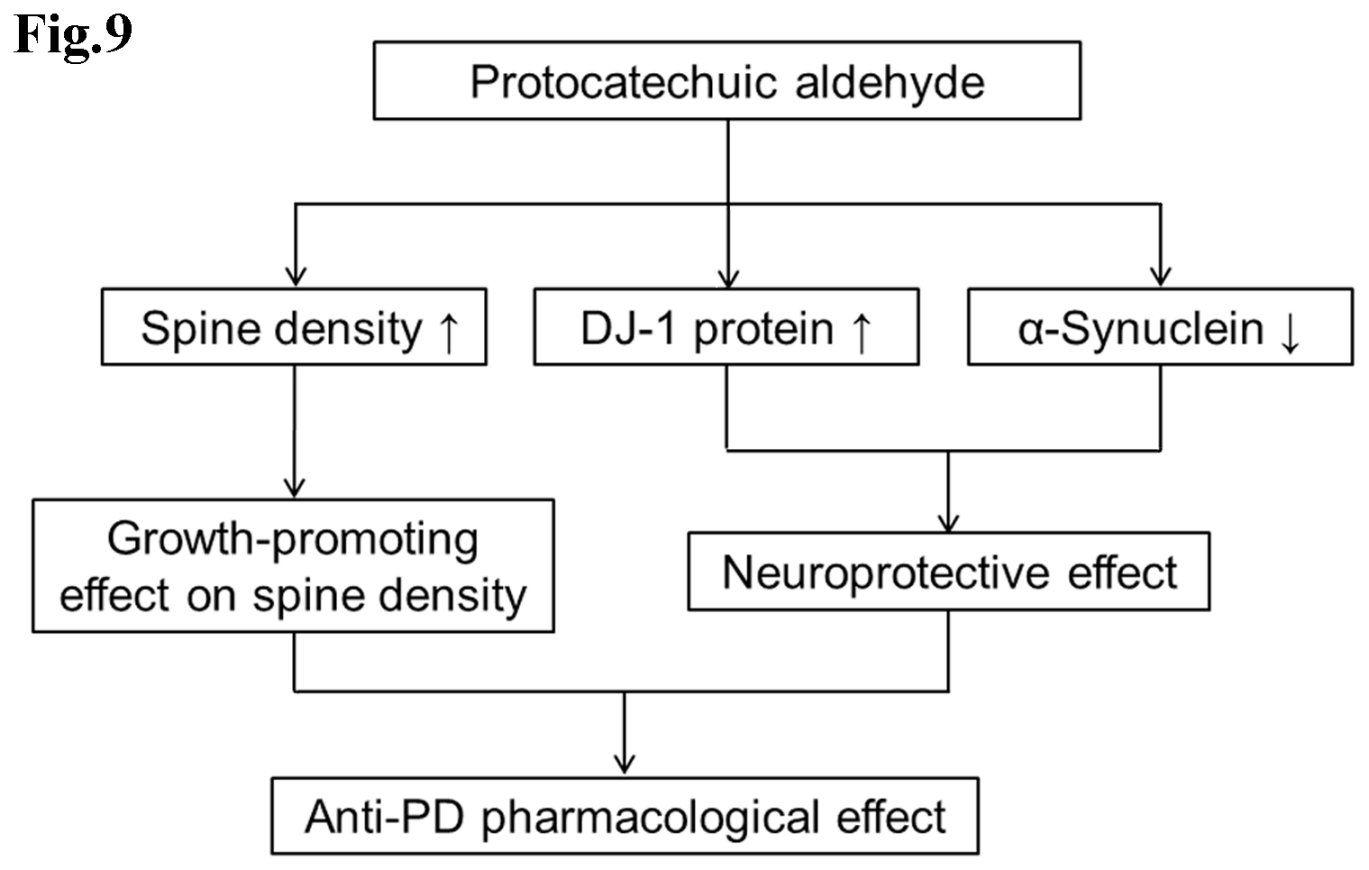
Proposed mechanisms by which PAL exerts anti-PD effects.

Taken together, two PD cell models induced by H_2_O_2_ or 6-OHDA in PC12 cells and two PD animal models, mice or rats model respectively induced by MPTP or 6-OHDA, were used to investigate the neuroprotective pharmacological effects of PAL. We have demonstrated that PAL can efficiently protect dopaminergic neurons against neurotoxin injury *in vitro* and *in vivo*. These results indicate that PAL is an important candidate compound for the treatment of PD. The potential mechanisms for the neuroprotective effects of PAL may be related to the increased level of DJ-1 and reduced level of α-synuclein, and in addition may also have a relationship with its growth-promoting effects on spine density (Figure 9). However, further studies are needed to investigate the detailed molecular mechanisms underlying the neuroprotective effects of PAL.
